# Supplementary material for: Cardiac health assessment across scenarios and devices using a multimodal foundation model pretrained on data from 1.7 million individuals
Source: Nat Mach Intell. 2026 Feb 24;8(2):220–33. doi: 10.1038/s42256-026-01180-5 (PMC12932102; doi:10.1038/s42256-026-01180-5)
Supplement: Supplementary file 1 — Supplementary dataset details, experimental settings and results. [file 42256_2026_1180_MOESM1_ESM.pdf]

nature machine intelligence

Supplementary information https://doi.org/10.1038/s42256-026-01180-5

Cardiac health assessment across scenarios and devices using a multimodal foundation model pretrained on data from 1.7 million individuals

In the format provided by the authors and unedited

S1 Dataset Curation Details

S1.1 Dataset details

Table S1 summarizes the datasets used for both pretraining and downstream tasks, encompassing over 2 million 10-second biosignal segments from more than 1.7 million unique individuals. Furthermore, the corresponding demographic information is provided in Table S2. These datasets cover a wide spectrum of acquisition settings, including intensive care units, operating rooms, primary care, telehealth, and home-based monitoring. Pretraining was conducted using major datasets collected primarily in the United States and Brazil, while downstream evaluations were performed across datasets from multiple countries worldwide.

We note that, despite the diversity of data sources, potential geographic or demographic biases may still exist, and the model's generalizability to underrepresented populations should not be assumed without further validation.

Table S1. Dataset characteristics, signal modalities, and associated downstream tasks. DIR: Demographic Information Recognition; CDD: Cardiovascular Disease Diagnosis; VSM: Vital Sign Measurement; COP: Clinical Outcome Prediction; QA: ECG Question Answering.

Dataset		ECG	PPG	Text	# Segments	# Individuals	DIR	CDD	VSM	COP	QA
Pretraining	MIMIC-III-WDB†	Lead II	✓	Partial	270,562	7,425	–	–	–	–	–
	MIMIC-IV-ECG†	12 Leads	✗	✓	787,677	160,821	–	–	–	–	–
	CODE-Full	12 Leads	✗	✗	1,558,748	1,558,748	–	–	–	–	–
Downstream	PTB-XL	12 Leads	✗	✗	21,388	18,562	–	✓	–	–	–
	SimBand	–	✓	✗	7,590	40	–	✓	–	–	–
	CinC17	Wearable	✗	✗	8,528	8,528	–	✓	–	–	–
	VitalDB	Lead II	✓	✗	574,800	1,409	✓	–	✓	–	–
	VTaC	Lead II	✓	✗	4,613	2,147	–	–	–	✓	–
	CODE-15	12 Leads	✗	✗	233,647	233,647	–	–	–	✓	–
	ECG-QA*	12 Leads	✗	✓	231,536	–	–	–	–	–	✓

*A version modified from PTB-XL.

†MIMIC IV and MIMIC III may include overlapping individuals but are de-identified and cannot be linked.

Table S2. Dataset country or region, acquisition scenario, and population demographics. Age is reported as median [interquartile range]; sex distribution is reported as the percentage of male participants. Statistics are calculated over all segments.

Dataset	Country/Region	Scenario	# Individuals	Age (years)	Sex (male, %)
MIMIC-III-WDB†	US	ICU	7,425	63 [52, 75]	56.0
MIMIC-IV-ECG†	US	Hospital / Clinical	160,821	66 [54, 77]	51.1
CODE-Full	Brazil	Primary Care / Telehealth	1,558,748	54 [41, 67]	39.7
PTB-XL	Germany	Hospital / Clinical	18,562	62 [50, 72]	52.1
SimBand	US	Simulated Ambulatory	40	-	-
CinC17	US	Ambulatory / Home	8,528	-	-
VitalDB	South Korea	Operating Room	1,409	61 [51, 70]	42.3
VTaC	US	ICU	2,147	-	-

† Ages above 89 years are truncated in MIMIC-III and MIMIC-IV in accordance with database de-identification policy.

S1.2 Dataset Specific

MIMIC-III-WDB. This dataset includes continuous bedside monitor recordings from ICU patients. We selected signals that contained both ECG Lead II and PPG waveforms, and parsed them into 10-second segments sampled at 12-hour intervals. Each segment was evaluated for signal quality using flat-line detection, skewness-based indices, and peak detection metrics. Segments that passed all quality checks were retained and subsequently processed using the preprocessing pipeline described above. The corresponding clinical text was extracted from the MIMIC-III Clinical Database and aligned to waveform segments within a 14-day temporal window, as described in the previous section.

MIMIC-IV-ECG. Within this dataset, we excluded recordings that contained NaN values or flat recordings. The ECG signals were subsequently preprocessed following the pipeline described above, and paired with their corresponding structured text reports.

CODE-FULL. The CODE-FULL dataset was preprocessed using the accompanying official scripts, and we selected only the first available recording for each unique individual. This was followed by the above preprocessing pipeline for standardization.

PTB-XL. PTB-XL contains 12-lead ECG recordings annotated with rich diagnostic metadata. We retained only recordings with non-empty diagnostic labels, as determined from the `scp_codes` field. Labels were aggregated using the associated `scp_statements.csv`, which maps SCP codes to all diagnostic classes (44 classes). They are highly imbalanced, with the minority class accounting for only 0.07% compared to the majority at 44.48%. Only entries marked as diagnostic were excluded. They were preprocessed using the pipeline, and the final processed dataset was used for downstream classification tasks involving multi-label cardiovascular disease prediction.

SimBand. This dataset contains wearable PPG recordings originally sampled at 50 Hz. Each segment was annotated with one of four rhythm categories: normal sinus rhythm (7.75%), atrial fibrillation (2.13%), premature atrial/ventricular contractions (1.66%), and noisy PPG (88.46%). Segments labeled as "NaN" due to insufficient reference ECG were excluded. They were preprocessed by our pipeline for standardization.

CinC17. The PhysioNet/CinC 2017 dataset comprises single-lead ECG recordings sampled at 300 Hz, labeled into four rhythm classes: normal sinus rhythm (59.22%), atrial fibrillation (8.65%), other rhythm (28.80%), and noisy recordings (3.33%). They were preprocessed into 10-second recordings following our pipeline. The resulting one-channel ECG segments and corresponding labels were saved in HDF5 format for use in downstream classification tasks.

VitalDB. This dataset includes ECG, PPG, and arterial blood pressure (ABP) waveform signals collected from bedside monitors in clinical settings. We used the curated version provided by PulseDB, in which 10-second windows were already extracted through window slicing and signal quality assessment. The waveform segments were further preprocessed following our standard pipeline. Each segment is accompanied by metadata including systolic and diastolic blood pressure (SBP and DBP), as well as patient demographics such as age, gender, and, where available, height, weight, and body mass index (BMI). For the vital sign measurement task, we adopted a calibration-based split strategy to simulate practical clinical deployment. For the demographic information recognition task, we used a calibration-free split to ensure there was no subject overlap between training, validation, and test sets.

VTaC. It consists of ECG and PPG waveform recordings, used to support binary classification of ventricular tachyarrhythmia (VT) events. From each record, we extracted a 10-second segment beginning at minute 5 of the recording (i.e., from 300 to 310 seconds) at a sampling rate of 250 Hz. Lead-II ECGs and PPG waveforms (PLETH, PLETH L/R) were required for inclusion. ECG signals were denoised using the `ecg_clean` function from NeuroKit2, while PPG signals underwent flat-line detection, skewness-based SQI assessment, and peak detection using HeartPy. PPG segments with insufficient peaks or irregular waveform morphology were excluded. Any NaNs were imputed with the channel-wise mean; recordings with remaining missing values were discarded. Signals passing all quality checks were standardized and stored as two-channel segments (ECG and PPG). Corresponding VT event labels were assigned using metadata files, and we followed the official split for benchmarking. The false alarm ratio is 27.33%.

CODE-15. This dataset contains 12-lead ECG recordings with structured demographic and clinical outcome labels. We used ECG signals from patients for whom both time-to-event (TTE) and event labels were explicitly reported (i.e., not inferred or missing). They were preprocessed, with the mortality label extracted as a binary classification task. The 1-year mortality rate is 1.23%.

ECG-QA. It is a multi-choice question answering dataset, and we chose its PTB-XL version. Each sample consists of a 10-second, 12-lead ECG tracing paired with a natural language question, its ground-truth answer (out of 103 templates), and a set of valid answer choices. We extracted training, validation, and test splits from the official ECG-QA release. Only entries with single-question-type labels (`question_type1` = 0) were retained. Answers were represented as 103-dimensional multi-hot vectors to support multi-label outputs.

S1.3 Preprocessing

In this section, we present details on how to derive segments in different datasets, especially for those long-term recordings.

S1.3.1 ECG

The raw ECG signals were first denoised using the `ecg_clean` function from NeuroKit2, followed by resampling and truncation/zero-padding to a fixed duration of 10 seconds (2,500 samples at 250 Hz). Signals were then standardized

using z-normalization, with normalization performed independently for each channel in multi-lead recordings. For long-term ECG recordings, window slicing strategies varied slightly across datasets, as detailed in Section S1.2.

S1.3.2 PPG

The raw PPG signals were denoised using the `ppg_clean` function from NeuroKit2, which applies bandpass filtering to reduce motion artifacts and baseline drift. Subsequently, each signal was resampled and truncated/zero-padded to a fixed duration of 10 seconds (2,500 samples at 250 Hz), and standardized using z-normalization. As with ECG, window slicing strategies for long-term recordings varied slightly across datasets and are described in Section S1.2.

S1.3.3 Text

The text information used for pretraining comes from two resources: **MIMIC-III-WDB** and **MIMIC-IV-ECG**.
MIMIC-III-WDB does not directly include cardiologist reports with its waveform recordings. We utilized the accompanying MIMIC-III Clinical Database[1] to retrieve and associate relevant textual interpretations (*i.e.*, ECG reports) with the ECG and PPG signals. These reports were aligned with waveform segments based on subject identifiers and approximate recording times, with a temporal window of up to 14 days to ensure plausibility.

Prior to use, reports were preprocessed to remove templated phrases (*e.g.*, "TRACING #X"), redundant headers, and extraneous punctuation. Text normalization and tokenization were performed using lightweight Python scripts (available in the Supplementary Code [*TBD*]), ensuring consistent formatting across reports.

Cleaned text was then paired with ECG and PPG recordings that passed basic signal quality checks, using toolkits such as NeuroKit2 and HeartPy to assess rhythm stability and peak detection. This process yielded a multi-modal dataset of curated biosignal–text pairs that incorporate clinical narratives alongside physiological signals. The preprocessing scripts are provided in the supplementary code repository. Although this form of "weak matching" may introduce limited noise, ablation experiments (Section S3.5) demonstrate the benefits of incorporating text data for biosignal representation learning.

An example mapping preprocessed text is included for reference. *"Normal sinus rhythm with atrio-ventricular conduction delay, Poor R wave, progression in leads V1-V3 consistent with possible old anteroseptal myocardial infarction"*
MIMIC-IV-ECG contains paired structured machine-generated textual reports. For each trace, we retrieved the signal file and the corresponding measurement summary from the `machine_measurements.csv` file, which contains multiple separate report fields per record. These fields were concatenated in order, separated by commas, to form a single composite diagnostic statement for each ECG recording. The preprocessing scripts are provided in the supplementary code.

An example text is included for reference. *"Sinus rhythm, Possible right atrial abnormality, Borderline ECG"*

On the downstream side, the text information is used in **ECG-QA**, where natural language questions serve as the input. These questions act as the textual prompts for the ECG-based Question Answering (QA) task during downstream evaluation.

S1.4 Evaluation Metrics

The evaluation metrics for the three groups of downstream tasks are summarized as follows.
Binary classification. For binary tasks, the primary evaluation metric was the area under the receiver operating characteristic curve (AUC), as it provides a threshold-independent measure of discriminative performance.
Multi-class and multi-label classification. For multi-class and multi-label tasks, the primary metric was the macro-averaged F1-score, which equally weights all classes and is less affected by label imbalance. For completeness, Matthews correlation coefficient (MCC) values are also reported in the Supplementary Table S5 for complementary evaluation. In particular, the ECG-QA task is formulated as a multi-label classification problem, where the model selects the correct answer(s) from a predefined set of candidate options. When computing the metrics, false positives outside the given candidates are not considered. Therefore, a modified version of the F1-score [1] was used to better reflect task-specific accuracy.

[1] `https://physionet.org/content/mimiciii`

Regression. For regression tasks, such as vital sign estimation, the mean absolute error (MAE) was used as the primary evaluation metric, as it is more interpretable and less sensitive to outliers than the root mean squared error (RMSE). The RMSE was additionally reported, in some cases, to provide complementary assessments.

All metrics were computed on held-out test sets using implementations from the `scikit-learn` library.

S2 Experimental Settings

S2.1 Domain Features

We extracted commonly used domain-specific features to serve as a baseline for comparison with features learned by CSFM. For ECG, we computed 54 handcrafted features per channel, capturing both morphological characteristics and heart rate variability. Feature extraction was based on R-peak detection and waveform delineation using NeuroKit2. For multi-lead ECGs, features from individual channels were averaged to obtain a consolidated representation. For PPG, we used pyPPG to extract a total of 306 features reflecting pulse morphology and variability. All feature extraction scripts are included in the supplementary code. For multi-lead/multi-modality settings, the features were extracted from each lead/modality respectively and then concatenated together. We further perform preliminary interpretability analysis of CSFM features by using these domain features in section S3.4.

Table S3. Comparison of handcrafted ECG and PPG feature groups. All features are organized into three interpretable physiological categories. Representative examples are shown with feature names and short descriptions.

Feature Group	ECG Features	PPG Features
**Interval-related features**	*Timing between cardiac events and intra-beat durations.*<br>Examples:<br>[RR0] previous R–R interval,<br>[t_PR] P–R interval,<br>[t_QT] Q–T interval,<br>[RR_m/1] average-to-current RR ratio.	*Pulse-to-pulse intervals, widths, and fiducial timings.*<br>Examples:<br>[Tpi] pulse onset-to-offset interval,<br>[Tpp] peak-to-peak interval,<br>[Tsp] systolic peak time,<br>[Tsw50] systolic width at 50% amplitude.
**Amplitude-related features**	*Waveform heights and voltage differences.*<br>Examples:<br>[a_R] R-peak amplitude,<br>[a_RS] R–S drop,<br>[a_ST/QS] repolarization-to-depolarization ratio,<br>[a_RS/QR] QRS symmetry index.	*Waveform magnitudes, amplitude ratios, and area-based measures.*<br>Examples:<br>[Asp] systolic peak amplitude,<br>[Adn] dicrotic notch amplitude,<br>[Adp] diastolic peak amplitude,<br>[AUCsys] area under the systolic segment.
**Other temporal / dynamic features**	*Beat-to-beat variability and signal quality.*<br>Examples:<br>[SDNN] standard deviation of NN intervals,<br>[RMSSD] root mean square of successive differences,<br>[TINN] triangular HRV index,<br>[ECG_SQI] signal quality index.	*Waveform dynamics, arterial stiffness, and composite indices.*<br>Examples:<br>[AI] augmentation index (Tp2–Tp1 difference / Asp),<br>[RIp1] reflection index at p1,<br>[RIp2] reflection index at p2,<br>[IPAD] inflection-point area + normalized d-point amplitude.

S2.2 Foundation Model Feature Extraction

To ensure fair comparison with CSFM, we extracted embeddings from several publicly available foundation models trained on time series or physiological signals. All models were used in their released pretrained form without

fine-tuning, as listed in Table S4.
ECG-FM [2] is a self-supervised foundation model pretrained on large-scale diagnostic ECGs using masked signal modeling. We employed the official weights pretrained on MIMIC-IV-ECG (`https://github.com/bowang-lab/ECG-FM`) and removed the final classification head for feature extraction. Segment-level embeddings were derived by mean-pooling the final hidden layer across temporal positions. This model was directly applied to 12-lead ECGs.
PaPaGei [3] is a foundation model pretrained on PPG signals from the VitalDB, MIMIC-III, and MESA [4] datasets. We followed the official tutorial (`https://github.com/nokia-bell-labs/papagei-foundation-model`) to process each 10-second PPG segment and extracted embeddings using the `papagei_s` version.
ECGFounder [5] was pretrained on over ten million ECG recordings from the Harvard–Emory Dataset (`https://bdsp.io/content/heedb/`). It provides two model versions (one for 12-lead and one for single-lead ECGs). We followed the official implementation (`https://github.com/NickLJLee/ECGFounder`) for feature extraction, using the 12-lead and single-lead versions separately.
MERL [6] was pretrained on MIMIC-IV ECG recordings. We followed the official repository (`https://github.com/cheliu-computation/MERL-ICML2024/`) and applied the `vit_tiny_best_encoder.pth` version for feature extraction. This model was applied to 12-lead ECG settings.
D-BETA [7] is a model pretrained on MIMIC-IV-ECG. We followed the official pipeline (`https://github.com/manhph2211/D-BETA`) to extract features, which were applied to 12-lead ECG recordings.
NormWear [8] is a foundation model that supports multivariate physiological signals as input. It was pretrained on multiple physiological datasets comprising ECG, PPG, galvanic skin response (GSR), etc. We followed the official pipeline (`https://github.com/Mobile-Sensing-and-UbiComp-Laboratory/NormWear`) to extract features from Lead-II ECG, PPG, or their combinations.
Chronos [9] is a general-purpose, encoder-only foundation model pretrained on large-scale time series data. We used its encoder to extract feature representations following the official implementation (`https://github.com/amazon-science/chronos-forecasting`). The feature extraction pipeline was consistent with that of PaPaGei[2], using the `chronos-t5-base` version. Since Chronos only supports univariate input (*i.e.*, single-channel time series), it was applied to Lead-II ECG or PPG signals separately.
Moment [10] is another open-source general time series foundation model, with repository online (`https://github.com/moment-timeseries-foundation-model/moment`). We used the `MOMENT-1-large` version and followed the feature extraction pipeline provided by PaPaGei[3]. Moment supports multivariate inputs, and we applied it to Lead-II ECG, PPG, and combined ECG–PPG configurations.

S2.3 Classical Machine Learning Models.

Following standard scaling of the input features, three types of classical machine learning models were applied to evaluate the discriminative quality of the extracted signal embeddings: logistic regression (`scikit-learn`), random forest (`scikit-learn`), and XGBoost (`xgboost package`). The logistic regression model used default settings with `max_iter=1000`. The random forest model was configured with `n_estimators=300` and the XGBoost model was configured with `n_estimators=300` and `max_depth=6`, and a learning rate of 0.05, while all other parameters remained at their default values. For regression tasks, the XGBoost regressor used the same configuration with `objective='reg:squarederror'` and `eval_metric='rmse'`.

Table S4. Overview of foundation models utilized in this study. Their originally supporting data type, pretrained ECG/PPG datasets, modality used in this work, and embedding dimensions are listed.

Model	Original Supporting Data Type	Pretrained ECG/PPG Datasets (if applicable)	Modality Used in This Study	Embedding Dim.
Chronos [9]	General univariate time series	–	Lead-II ECG, PPG (univariate)	768
Moment [10]	General multivariate time series	–	Lead-II ECG, PPG, ECG–PPG	1024
NormWear [8]	Wearable multivariate physiological data	Multiple physiological datasets (ECG, PPG, GSR, etc.)	Lead-II ECG, PPG, Lead-II ECG+PPG	768
PaPaGei [3]	PPG	VitalDB, MIMIC-III, MESA	PPG	512
ECG-FM [2]	12-Lead ECG	MIMIC-IV-ECG	12-lead ECG	768
ECGFounder [5]	12-Lead ECG, Single-Lead ECG (two versions)	Harvard–Emory	12-lead, single-lead ECG	1024
D-BETA [7]	12-Lead ECG	MIMIC-IV-ECG	12-lead ECG	768
MERL [6]	12-Lead ECG	MIMIC-IV-ECG	12-lead ECG	192

[2] `https://github.com/Nokia-Bell-Labs/papagei-foundation-model/blob/main/linearprobing/feature_extraction_chronos.py`
[3] `https://github.com/Nokia-Bell-Labs/papagei-foundation-model/blob/main/linearprobing/feature_extraction_moment.py`

S2.4 Prompt Template for LLaVA

We used LLaMA3-LLaVA-Next-8B as the "baseline" for ECG-QA results, available in `https://huggingface.co/lmms-lab/llama3-llava-next-8b`. An example of the prompt tailored for ECG-QA is shown in Figure S1.

Prompt template

You are a medical assistant reviewing a 12-lead ECG plot.

QUESTION:
Which diagnostic symptom does this ECG show, non-specific ST changes or myocardial infarction in inferoposterolateral leads, excluding uncertain symptoms?

Please choose the correct answer from the following options:
A. myocardial infarction in inferoposterolateral leads
B. non-specific ST changes

You may choose zero, one, or more options. Reply with the letters of all applicable answers, separated by commas (*e.g.*, "A, C, E").

Answer:

Figure S1. Prompt template of LLaVA for ECG-QA.

Table S5. Performance comparison of different methods across three ECG datasets (PTB-XL, CinC17, and SimBand). Performance was measured using Macro-F1 and MCC metrics (mean [95% CI] across three random seeds). XGB prediction based on domain features is also listed for benchmarking.

Method	SimBand		CinC17		PTB-XL	
	Macro-F1	MCC	Macro-F1	MCC	Macro-F1	MCC
Domain Feature	0.328 [0.308, 0.348]	0.360 [0.311, 0.410]	0.496 [0.470, 0.523]	0.393 [0.363, 0.424]	0.258 [0.256, 0.260]	0.244 [0.238, 0.251]
BiLSTM	0.332 [0.302, 0.364]	0.332 [0.306, 0.358]	0.588 [0.540, 0.636]	0.455 [0.370, 0.540]	0.288 [0.260, 0.315]	0.293 [0.260, 0.327]
ResNet1d18	0.357 [0.324, 0.390]	0.435 [0.338, 0.532]	0.606 [0.557, 0.655]	0.484 [0.386, 0.583]	0.324 [0.307, 0.342]	0.323 [0.303, 0.344]
ResNet1d34	0.334 [0.308, 0.360]	0.329 [0.227, 0.431]	0.634 [0.558, 0.710]	0.557 [0.451, 0.662]	0.328 [0.296, 0.361]	0.325 [0.278, 0.373]
ResNet1d50	0.347 [0.341, 0.353]	0.354 [0.278, 0.429]	0.561 [0.495, 0.627]	0.496 [0.439, 0.553]	0.322 [0.317, 0.328]	0.319 [0.306, 0.332]
ResNet1d101	0.351 [0.297, 0.405]	0.378 [0.147, 0.609]	0.592 [0.508, 0.675]	0.518 [0.465, 0.572]	0.326 [0.304, 0.347]	0.321 [0.300, 0.341]
Inception1d	0.344 [0.323, 0.365]	0.337 [0.262, 0.411]	0.601 [0.517, 0.684]	0.462 [0.386, 0.537]	0.323 [0.285, 0.361]	0.317 [0.284, 0.350]
MSDNN	0.337 [0.315, 0.360]	0.321 [0.199, 0.442]	0.618 [0.570, 0.666]	0.537 [0.466, 0.608]	0.294 [0.268, 0.320]	0.283 [0.259, 0.307]
CSFM-Tiny	0.354 [0.338, 0.370]	0.384 [0.346, 0.421]	0.656 [0.646, 0.667]	0.575 [0.542, 0.608]	0.342 [0.309, 0.374]	0.342 [0.313, 0.372]
CSFM-Base	0.354 [0.314, 0.395]	0.417 [0.283, 0.550]	0.655 [0.617, 0.694]	**0.631 [0.609, 0.653]**	**0.357 [0.338, 0.377]**	0.373 [0.364, 0.383]
CSFM-Large	**0.398 [0.279, 0.516]**	**0.439 [0.405, 0.473]**	**0.677 [0.656, 0.698]**	0.620 [0.568, 0.671]	0.338 [0.314, 0.362]	**0.412 [0.396, 0.429]**

S3 Supplementary Results

S3.1 Results of Additional Metrics

To provide a complementary assessment to the F1-score, we report the Matthews correlation coefficient (MCC) across three ECG datasets (SimBand, CinC17, and PTB-XL), supplemented by domain-feature based XGBoost prediction, and BiLSTM. As shown in Supplementary Table S5, the trends in MCC are largely consistent with those observed for Macro-F1, further confirming the robustness of the performance comparison.

Among the baseline models, ResNet1d18-101 and MSDNN achieve moderate performance, with Macro-MCC values typically ranging between 0.28 and 0.55 across datasets. In contrast, the proposed CSFM models consistently outperform these baselines.

Specifically, CSFM-Large achieves the highest MCC on SimBand (0.439 [0.405, 0.473]) and PTB-XL (0.412 [0.396, 0.429]), indicating stronger generalization across both wearable and clinical ECG domains. On the CinC17 dataset, CSFM-Base attains the best MCC (0.631 [0.609, 0.653]), reflecting stable discriminative capability across

cardiac conditions. These findings align with the Macro-F1 results, demonstrating that larger CSFM variants provide consistent gains over conventional convolutional architectures.

S3.2 Additional Results of PTB-XL

PTB-XL can also be grouped into 5 superclass categories, *i.e.*, Normal (NORM), Myocardial Infarction (MI), ST/T Change (STTC), Hypertrophy (HYP), and Conduction Disturbance (CD)), representing broader diagnostic groups. To further examine model generalization under different label granularities, we conducted additional experiments on both the 5-class superclass and the full 44-class diagnostic settings, based on XGBoost.

As shown in Supplementary Table S6, the trends in performance between the two settings are consistent. When evaluated under the coarser 5-class configuration, all models achieved substantially higher F1 and MCC scores, reflecting the reduced label complexity. Across both configurations, CSFM variants achieved competitive or superior performance compared with existing foundation models, particularly in the 44-class setting, where CSFM-Base attained the highest MCC (0.330 [0.315, 0.346]) and CSFM-Large achieved the best F1 (0.331 [0.316, 0.346]). These findings highlight the robustness of CSFM representations in multi-label ECG classification tasks of varying difficulty.

Table S6. Comparison between 5-class superclass and 44-class PTB-XL diagnostic settings. All values are mean [95% CI] across three random seeds. **Bold** indicates best overall performance.

Model	Superclass (5 Classes)		Main Paper Settings (44 Classes)	
	Macro-F1	MCC	Macro-F1	MCC
Domain Feature	0.555 [0.548, 0.562]	0.393 [0.387, 0.399]	0.258 [0.256, 0.260]	0.244 [0.238, 0.251]
ECG-FM	0.537 [0.529, 0.545]	0.392 [0.383, 0.401]	0.267 [0.262, 0.271]	0.254 [0.248, 0.259]
D-BETA	0.563 [0.555, 0.572]	0.435 [0.425, 0.445]	0.231 [0.223, 0.239]	0.235 [0.206, 0.263]
MERL	0.591 [0.586, 0.597]	0.445 [0.440, 0.451]	0.273 [0.264, 0.282]	0.266 [0.258, 0.274]
ECGFounder	**0.618** [0.610, 0.626]	**0.505** [0.497, 0.513]	0.310 [0.304, 0.316]	0.310 [0.297, 0.323]
CSFM-Tiny	0.604 [0.602, 0.606]	0.470 [0.463, 0.477]	0.309 [0.287, 0.330]	0.304 [0.278, 0.329]
CSFM-Base	0.596 [0.590, 0.602]	0.469 [0.461, 0.477]	0.327 [0.321, 0.333]	**0.330** [0.315, 0.346]
CSFM-Large	0.604 [0.603, 0.606]	0.480 [0.474, 0.487]	**0.331 [0.316, 0.346]**	0.323 [0.308–0.339]

Across both settings, ECGFounder exhibited the strongest overall performance among existing foundation models, likely benefiting from extensive pretraining on large-scale ECG data and diagnostic label association during pretraining [5]. Future work may focus on incorporating diagnostic-specific priors and expanding pretraining to even larger clinical biosignal corpora, which may further narrow the gap with task-optimized foundation models such as ECGFounder.

S3.3 Additional Results of VitalDB

Additional benchmarking on demographic information recognition is shown in Figure S2, extending the analyses in Figures 3 and 4 of the main text. In particular, Figure S2a presents the performance of CSFM and non-CSFM models on age, gender, and BMI prediction under different input-channel configurations (ECG-only, PPG-only, and combined ECG+PPG). Figure S2b further compares the performance based on domain features, foundation-model-derived features, and CSFM-derived features across the same tasks. Across all tasks, fine-tuned CSFM variants consistently outperformed directly trained deep models, with the combined-channel configuration yielding the best overall performance. It is also noteworthy that PPG signals performed relatively better than Lead-II ECG for age prediction, but worse for gender and BMI estimation. Future analyses on larger and more diverse cohorts may further validate this observation and provide deeper insights into the comparative predictive competence of ECG and PPG modalities.

S3.4 Correlation between Domain Features and Foundation Model Derived Features

To investigate the relationship between physiological features and learned latent representations, we conducted a correlation analysis between the top domain-engineered features and the principal components (PCs) of the foundation-model embeddings. We trained an XGBoost classifier on handcrafted domain features to identify the top ten most informative features for the downstream classification task, as shown in Figure S3. Feature importance was determined using

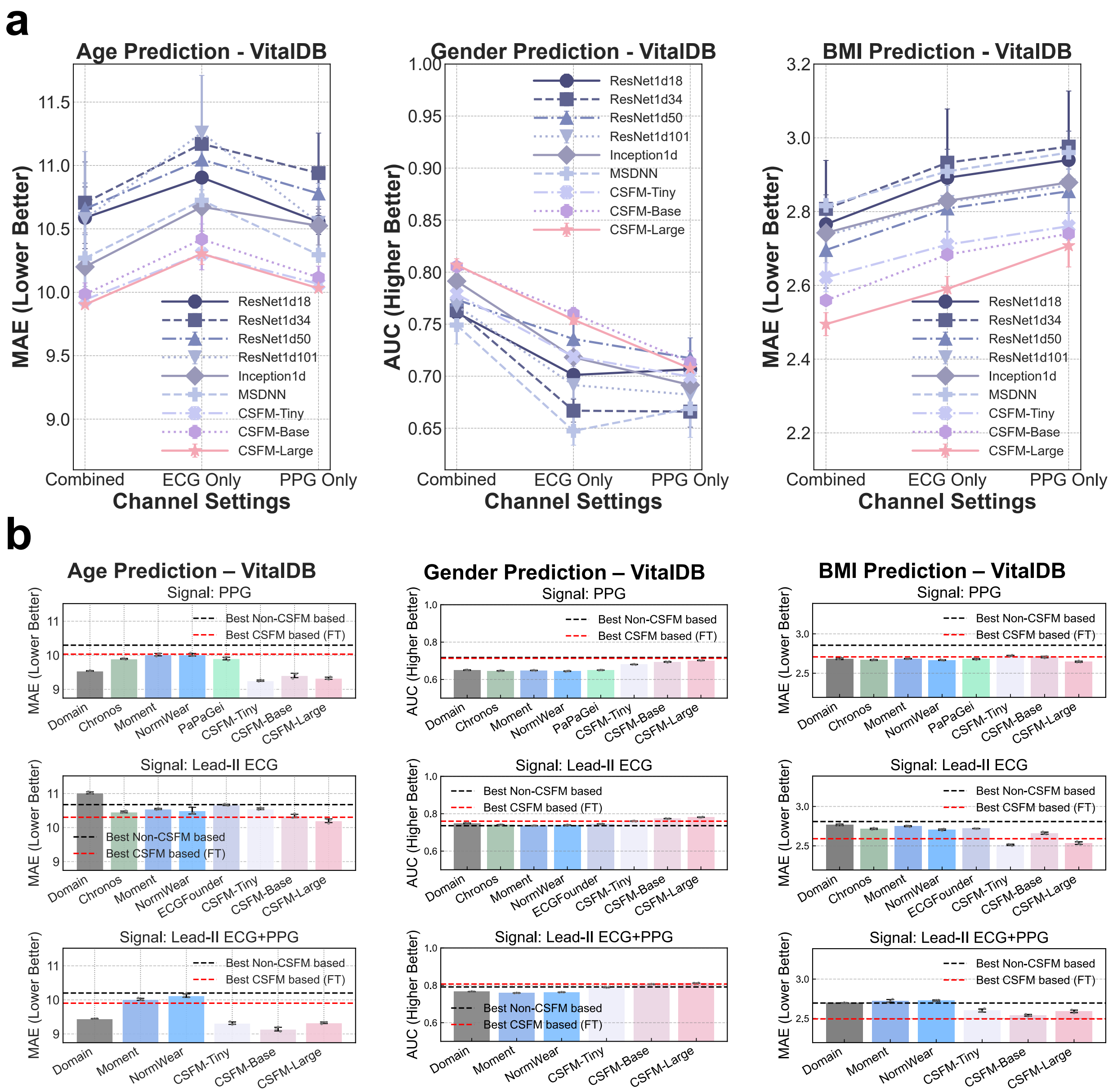

Figure S2. Additional results on VitalDB. a. Age, gender, and BMI prediction performance under different input-channel configurations (Lead-II ECG-only, PPG-only, or combined), comparing fine-tuned CSFM models with directly trained non-CSFM deep models. **b.** Performance comparison based on domain features, foundation-model-derived features, and CSFM-derived features across age, gender, and BMI prediction tasks, based on XGBoost, and reference lines indicate the best-performing CSFM-based and non-CSFM-based methods, reported in subfigure **a**.

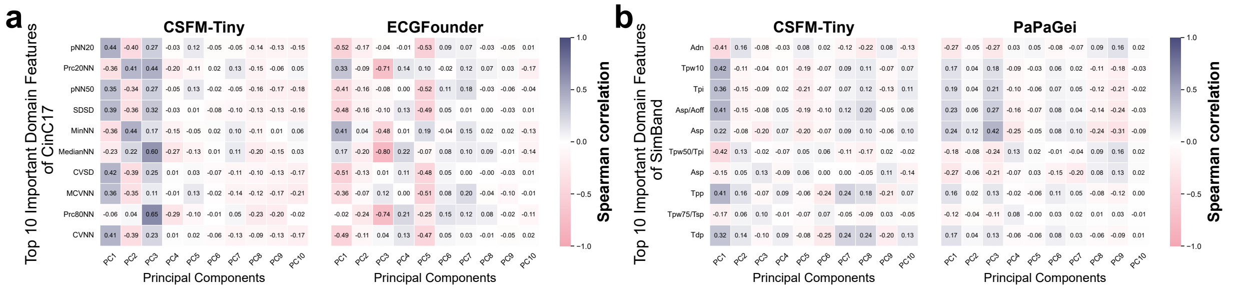

Figure S3. Spearman correlation between the top 10 most informative domain features and the first 10 principal components (PCs) of foundation-model embeddings. Blue indicates positive correlation, red indicates negative correlation. The analysis links interpretable physiological descriptors (rows) with latent representation axes (columns) for ECG (leftmost two) and PPG (rightmost two) modalities. *pNN20/pNN50*: percentage of successive RR differences > 20 / 50 ms; *SDSD*: standard deviation of successive RR interval differences; *CVSD/CVNN/MCVNN*: coefficients of variation of RR intervals or their differences; *Prc20NN/Prc80NN*: 20th/80th percentiles of RR intervals; *MedianNN/MinNN*: central and minimal RR intervals; *Adn*: dicrotic-notch amplitude; *Tpi*: pulse interval between consecutive pulse onsets; *Tpw10/Tpw50/Tpw75*: pulse-width durations at 10%, 50%, 75% amplitude; *Asp/Asp/Aoff*: systolic amplitude and its ratio to baseline; *Tpp*: peak-to-peak interval; *Tpw50/Tpi/Tpw75/Tsp*: ratios of width to interval or systolic-time, capturing waveform symmetry and arterial stiffness; *Tdp*: diastolic-peak time.

gain-based importance scores averaged across trees. Meanwhile, the 768-dimensional embeddings from each foundation model (CSFM-Tiny, ECGFounder, or PaPaGei) were standardized and projected onto their first ten principal components (PCs) using Principal Component Analysis (PCA). These PCs capture the major directions of variance in the embedding space. For each dataset, we computed Spearman's rank correlation coefficient between the ten selected domain features and the ten PCA components. The resulting 10×10 correlation matrices quantify how strongly each interpretable physiological variable aligns with individual embedding dimensions. Each cell in the heatmap corresponds to the correlation between a domain feature (row) and a PCA component (column).

Positive correlations (blue) indicate that a given PCA direction increases monotonically with a physiological measure (*e.g.*, heart rate variability indices such as pNN50 or RMSSD), while negative correlations (red) suggest an inverse relationship. The magnitude of the correlation value reflects the strength of alignment between an interpretable domain feature and a latent axis of the embedding space.

For ECG (CinC17), the first few PCs of both foundation models correlated most strongly with HRV-related features (pNN20, pNN50, SDNN, CVNN), suggesting that the models implicitly encode temporal rhythm variability without explicit supervision. For PPG (SimBand), the top PCs correlated with morphological features such as Adn (dicrotic notch amplitude), Tpi (pulse interval), and Asp/Aoff (systolic-to-onset amplitude ratio), reflecting sensitivity to waveform shape and vascular stiffness.

This line of analysis could serve as a valuable direction for improving the interpretability of the "black-box" foundation-model-derived features and for evaluating their intermediate representations. We consider this a promising avenue for future exploration.

S3.5 Ablation Study Results of Heterogeneous Pretraining

The pretraining of our foundation model leverages vast amounts of heterogeneous health records aggregated from multiple datasets. We performed a series of ablation experiments examining how different data configurations influence downstream performance.

Unified heterogeneous pretraining facilitates representation learning across leads. First of all, we conducted comparative experiments using two alternative pretraining strategies: one utilizing only the MIMIC-IV-ECG dataset and the other restricting the data to common channels (specifically, Lead-II ECG only). These represent "straightforward" solutions when dealing with heterogeneous datasets, either by relying on just a single relatively large dataset or by selecting only the overlapping modalities.

We benchmarked performance across different lead configurations on PTB-XL for these two ablated versions. As shown in Figure S4a, our foundation model outperforms both alternative training strategies in most cases. Notably, in 1-lead settings, the model pretrained on MIMIC-IV (which includes both texts and 12-lead ECGs) outperforms the model trained on Lead-II ECG only (which aggregates Lead-II ECGs across datasets). This finding suggests that integrating data from multiple modalities can yield better performance, even when it compromises overall dataset size.

Non-PPG modalities facilitate PPG representation learning. Furthermore, it is noteworhty that during pretraining only MIMIC-III-WDB includes PPG dataset, comprising approximately 270k PPG segments (7k individuals). We conducted experiments to validate whether additional modalities from ECG, associated clinical text, and even non-paired 12-lead ECGs/texts can facilitate the pretraining of a model that learns better representations of PPG. The results in Figure S4b show that models pretrained with a greater diversity of modalities achieve progressively better performance across five PPG-related tasks, demonstrating that richer multimodal supervision leads to more informative representations.

"Weak Match" facilitates ECG/PPG representation learning. On the other hand, it is noted that the weak matching approach, as mentioned in Section S1.3.3, may include information that is not immediately paired in the text and current biosignal snapshot. The results in Figure S4b compare pretraining on MIMIC-III PPG+ECG with on MIMIC-III PPG+ECG+text; the latter achieves better or comparable performance across five tasks, which demonstrates the importance of such "weak" text associations. This approach is particularly valuable in real-world settings, where exact matching between text and biosignals is often not achievable.

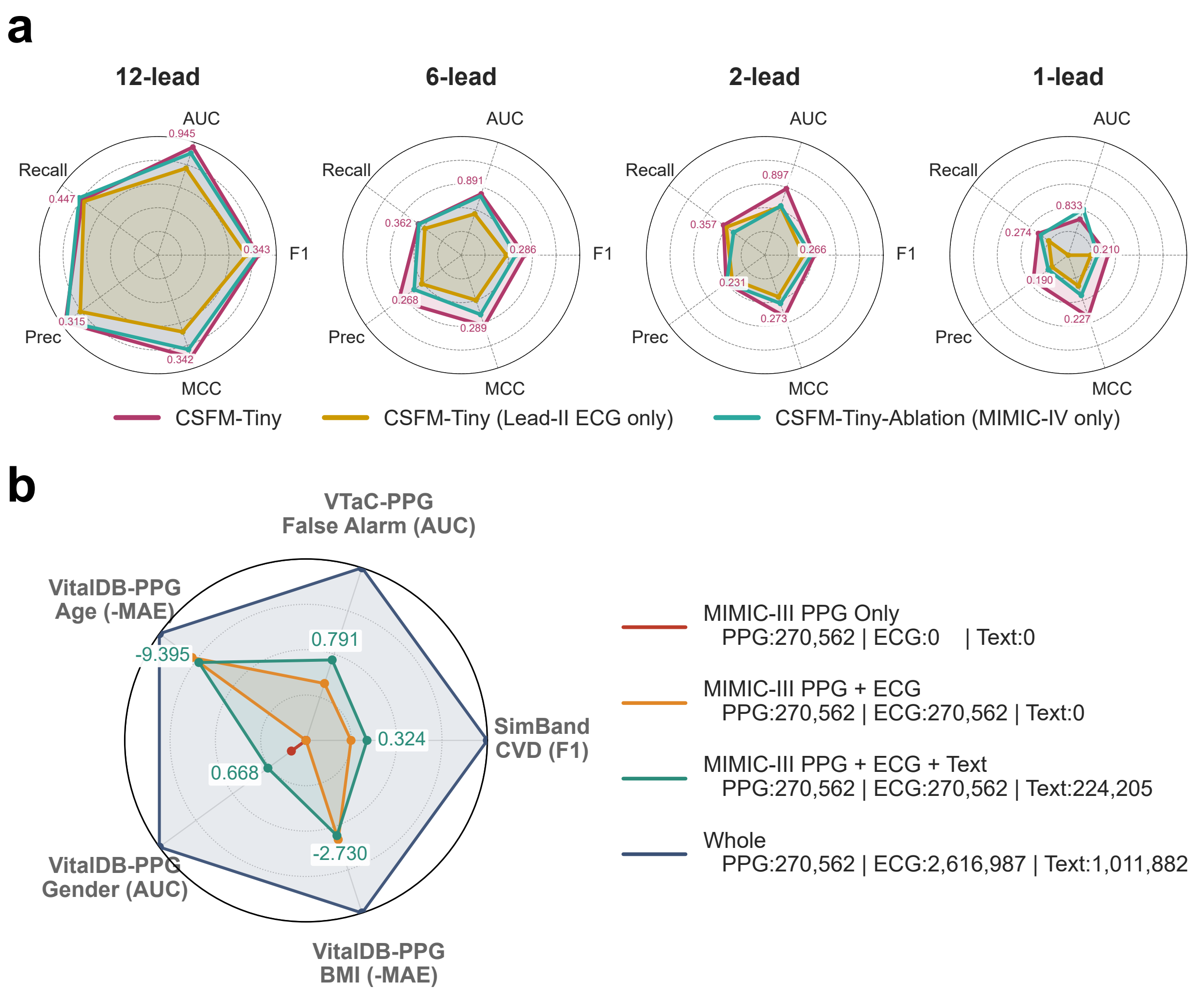

Figure S4. Ablation study for different pretraining settings. a. Comparison of our training strategies against other "straightforward" solutions to handling heterogeneous health records, (i) keeping the common channels only, *i.e.*, Lead-II ECG, (ii) keeping one dataset only, *i.e.*, MIMIC-IV including both 12-Lead ECGs and texts. Based on our training strategies and these two compared strategies, we assessed the performance disparity across varied lead settings on PTB-XL datasets. The radar axes in the figure are log-normalized and use the same range for better visualization. **b.** Comparison of pretraining strategies under different lead modalities and dataset combinations. Ablation models pretrained with (i) MIMIC-III PPG-only, (ii) MIMIC-III PPG+ECG, (iii) MIMIC-III PPG+ECG+Text, and (iv) the full heterogeneous pretraining set were compared across five PPG-related tasks. Performance values were min-max normalized within each task for clearer comparison.

S3.6 Additional Results of PPG-to-ECG Cross-Modality Reconstruction

We observed a clear performance gap between the train-on-synthetic, test-on-real and train-on-real, test-on-synthetic configurations, particularly in PPG-to-ECG cross-modality reconstruction. This asymmetry likely arises from these factors: (i) Although CinC17 and SimBand have similar segment sizes of around 8k, CinC17 contains 8,528 subjects, whereas SimBand includes only 40 individuals, thus constraining the diversity and representativeness of the synthetic domain. (ii) PPG signals (and occasionally ECG) are inherently noisy, and the use of a one-to-one reconstruction objective, such as mean squared error (MSE), can lead the model to learn overly smoothed waveforms that fail to capture real-world artifacts and the diverse physiological associations between PPG and ECG. Previous findings in the computer vision domain [11] have shown that MSE-based generative optimization tends to produce over-smoothed results with limited diversity, motivating the development of more advanced generative approaches such as adversarial, diffusion, or variational models.

Consequently, classification models trained purely on synthetic signals may not generalize well to the noise and variability present in real-world datasets. As a result, models trained on CinC17 can generalize reasonably well to synthetic data, whereas models trained exclusively on synthetic signals fail to generalize effectively to CinC17. Representative examples are provided in Figure S5b.

We introduced data augmentation strategies during synthetic pretraining, specifically temporal dropout, random scaling, and Gaussian noise, as shown in Figure S5a. These augmentations yielded measurable improvements and partially reduced the performance gap, although some of these gains may stem from the augmentations themselves.

On the other hand, we also highlight the need for more robust reconstruction objectives that go beyond simple MSE-based one-to-one matching and can better accommodate the inherent noise and diversity of real-world wearable signals. Nevertheless, the proposed CSFM framework can serve as a strong backbone for such developments. Future work may integrate diffusion-based objectives [12] and other generative formulations to further enhance the realism and variability of cross-modal reconstructions.

a

Methods	Waveform Reconstruction		Train-Real Test-Synthetic		Train-Syn (w/o Aug) Test-Real		Train-Syn (w Aug) Test-Real	
	MAE	RMSE	F1	AUC	F1	AUC	F1	AUC
UNet1d	0.608	0.964	0.427	0.591	0.276	0.558	0.453	0.534
BiLSTM	0.607	0.953	0.536	0.648	0.340	0.622	**0.590**	0.677
AutoEncoder[13]	0.585	0.927	0.442	0.784	0.353	0.600	0.420	0.607
CSFM-Tiny	0.532	0.863	0.690	0.812	**0.365**	0.669	0.570	**0.749**
CSFM-Base	0.524	0.852	0.632	0.815	0.364	**0.690**	**0.590**	0.703
CSFM-Large	**0.516**	**0.840**	**0.692**	**0.820**	0.353	0.688	0.585	0.731

b

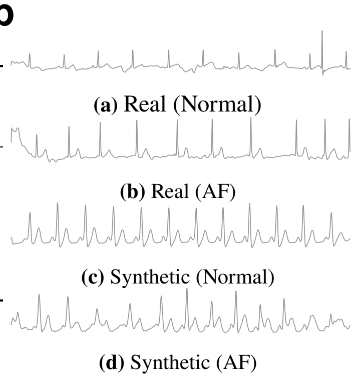

Figure S5. Cross-modality reconstruction and augmentation results. a. PPG to ECG Reconstruction. (1) The reconstruction was performed on VitalDB, and the waveform reconstruction performance was reported on the held-out test set of VitalDB. (2) Subsequently, we applied the adapted model to the original SimBand dataset to generate synthetic Lead-II ECG waveforms. To comprehensively test the quality of generated ECG waveforms, we conducted two experimental settings: train on synthetic ECG from SimBand (normal versus AF), and test on real ECG on CinC17 (normal versus AF), and vice versa, with an independent model ResNet1d18. The performance is reported using F1 and AUC. **Best** values are in bold, and second best are underlined. **b.** Examples of real and synthetic ECG examples belonging to normal and AF.

S3.7 Parameters and Computational Cost of CSFMs

The details of CSFM parameters are provided in Table S7. We acknowledge that the computational cost is non-negligible for foundation models per se, which represents a limitation of the current implementation.

The CSFM family ranges from the relatively lightweight *CSFM-Tiny* to the full *CSFM-Large*, maintaining a balanced trade-off between parameter count, computational efficiency, and performance.

Table S7. Details of our cardiac sensing foundation models. Att.: Attention; Enc.: Encoder. FLOPs are measured using a single-lead 10-second (250Hz) input segment, and also reported relative to ResNet1d18.

**Model scale**	**# Parameters**	**Representation size**		**Transformer block**		**FLOPs (×ResNet1d18)**
		Hidden	Intermediate	Att. head	#Enc. layer	
CSFM-Tiny	51M	1024	768	8	6	0.86G (4.4x)
CSFM-Base	117M	3072	768	12	12	3.83G (19.9x)
CSFM-Large	343M	4096	1024	24	16	61.07G(70.6x)

Furthermore, in the context of VTaC ICU deterioration events detection, which necessitate real-time prediction, CSFM demonstrates strong predictive performance (AUC over 0.7) even five minutes before alarm onset, as shown in Figure 4a of the main text. This highlights its potential applicability in real-time patient monitoring scenarios.

Nevertheless, optimizing CSFM for low-latency and resource-constrained environments remains an important direction for future work, particularly for deployment on wearable or embedded healthcare devices.

References

[1] Oh, J., Lee, G., Bae, S., Kwon, J.-m. & Choi, E. Ecg-qa: A comprehensive question answering dataset combined with electrocardiogram. In Oh, A. *et al.* (eds.) *Advances in Neural Information Processing Systems*, vol. 36, 66277–66288 (Curran Associates, Inc., 2023). URL https://proceedings.neurips.cc/paper_files/paper/2023/file/d0b67349dd16b83b2cf6167fb4e2be50-Paper-Datasets_and_Benchmarks.pdf.

[2] McKeen, K. *et al.* Ecg-fm: An open electrocardiogram foundation model. *arXiv preprint arXiv:2408.05178* (2024).

[3] Pillai, A., Spathis, D., Kawsar, F. & Malekzadeh, M. Papagei: Open foundation models for optical physiological signals. *arXiv preprint arXiv:2410.20542* (2024).

[4] Zhang, G.-Q. *et al.* The national sleep research resource: towards a sleep data commons. *Journal of the American Medical Informatics Association* **25**, 1351–1358 (2018).

[5] Li, J. *et al.* An electrocardiogram foundation model built on over 10 million recordings. *NEJM AI* **2**, AIoa2401033 (2025).

[6] Liu, C. *et al.* Zero-shot ecg classification with multimodal learning and test-time clinical knowledge enhancement. In *International Conference on Machine Learning*, 31949–31963 (PMLR, 2024).

[7] Hung, M. P., Saeed, A. & Ma, D. Boosting masked ecg-text auto-encoders as discriminative learners. In *Forty-second International Conference on Machine Learning*.

[8] Luo, Y., Chen, Y., Salekin, A. & Rahman, T. Toward foundation model for multivariate wearable sensing of physiological signals (2024). URL https://arxiv.org/abs/2412.09758. 2412.09758.

[9] Ansari, A. F. *et al.* Chronos: Learning the language of time series. *Transactions on Machine Learning Research* .

[10] Goswami, M. *et al.* Moment: A family of open time-series foundation models. In *International Conference on Machine Learning*, 16115–16152 (PMLR, 2024).

[11] Ledig, C. *et al.* Photo-realistic single image super-resolution using a generative adversarial network. In *Proceedings of the IEEE conference on computer vision and pattern recognition*, 4681–4690 (2017).

[12] Shome, D., Sarkar, P. & Etemad, A. Region-disentangled diffusion model for high-fidelity ppg-to-ecg translation. In *Proceedings of the AAAI conference on artificial intelligence*, vol. 38, 15009–15019 (2024).

[13] Gu, X., Guo, Y., Deligianni, F., Lo, B. & Yang, G.-Z. Cross-subject and cross-modal transfer for generalized abnormal gait pattern recognition. *IEEE Transactions on Neural Networks and Learning Systems* **32**, 546–560 (2020).
